# Location of Dual Sites in *E. coli* FtsZ Important for Degradation by ClpXP; One at the C-Terminus and One in the Disordered Linker

**DOI:** 10.1371/journal.pone.0094964

**Published:** 2014-04-10

**Authors:** Jodi L. Camberg, Marissa G. Viola, Leslie Rea, Joel R. Hoskins, Sue Wickner

**Affiliations:** 1 Laboratory of Molecular Biology, National Cancer Institute, National Institutes of Health, Bethesda, Maryland, United States of America; 2 Department of Cell and Molecular Biology, College of Environment and Life Sciences, University of Rhode Island, Kingston, Rhode Island, United States of America; University of Groningen, Groningen Institute for Biomolecular Sciences and Biotechnology, Netherlands

## Abstract

ClpXP is a two-component ATP-dependent protease that unfolds and degrades proteins bearing specific recognition signals. One substrate degraded by *Escherichia coli* ClpXP is FtsZ, an essential cell division protein. FtsZ forms polymers that assemble into a large ring-like structure, termed the Z-ring, during cell division at the site of constriction. The FtsZ monomer is composed of an N-terminal polymerization domain, an unstructured linker region and a C-terminal conserved region. To better understand substrate selection by ClpXP, we engineered FtsZ mutant proteins containing amino acid substitutions or deletions near the FtsZ C-terminus. We identified two discrete regions of FtsZ important for degradation of both FtsZ monomers and polymers by ClpXP in vitro. One region is located 30 residues away from the C-terminus in the unstructured linker region that connects the polymerization domain to the C-terminal region. The other region is near the FtsZ C-terminus and partially overlaps the recognition sites for several other FtsZ-interacting proteins, including MinC, ZipA and FtsA. Mutation of either region caused the protein to be more stable and mutation of both caused an additive effect, suggesting that both regions are important. We also observed that in vitro MinC inhibits degradation of FtsZ by ClpXP, suggesting that some of the same residues in the C-terminal site that are important for degradation by ClpXP are important for binding MinC.

## Introduction

AAA+ ATPases (ATPases associated with various cellular activities) represent a superfamily of ATPases that are present across kingdoms and encompass a variety of cellular functions, including intracellular trafficking, DNA replication, cytokinesis, protein folding and degradation. In *Escherichia coli*, the AAA+ ATPase ClpX partners with ClpP, a serine protease, to form a two-component ATP-dependent proteolytic machine. The substrate recognition component of the ClpXP protease is ClpX, which is present as a hexameric ring. Hexameric ClpX associates with ClpP, a barrel-shaped structure composed of two seven-membered rings with an internal proteolytic chamber. Using the energy from ATP hydrolysis, ClpX unfolds polypeptides and threads the polypeptide chain through the central channel of ClpX and into the central proteolytic chamber of ClpP where degradation occurs [1]. The N-domain of ClpX interacts with several substrates directly, including *E. coli* UmuD and bacteriophage proteins MuA and lambda O protein [2]–[4]. Some degradation substrates require an adaptor protein for efficient recognition. Adaptor proteins bind specifically to a substrate and to ClpX to promote substrate engagement and initiation of unfolding by ClpX [5]. For example, the SspB protein enhances degradation of ssrA-tagged substrates by promoting an interaction between ClpX and the ssrA-tag [6].

We previously demonstrated that ClpXP degrades polymerized and non-polymerized FtsZ in vitro, and the rate of degradation for polymerized FtsZ is faster than non-polymerized FtsZ [7]. FtsZ is recognized by ClpX directly and does not require an adaptor protein, however the N-domain of ClpX is important for degradation of FtsZ [7]. FtsZ, a homolog of the eukaryotic protein tubulin, is essential in *E. coli* and forms a large structure with ring-like architecture at the nascent division site; this structure is referred to as the Z-ring [8]. Formation of the Z-ring precedes constriction at the septum and cell separation. Evidence suggests that the Z-ring may be comprised of dynamic FtsZ protein filaments that are bundled and tethered to the inner face of the cytoplasmic membrane through direct interactions with membrane-associated proteins FtsA and ZipA [8].

FtsZ is a GTPase and assembles into dynamic polymers in the presence of GTP in vitro [9]. Several proteins in *E. coli* bind to FtsZ and influence the dynamic assembly of FtsZ fibers [8]. These include ZipA, ZapA and ZapC, which stabilize FtsZ polymers from disassembly and promote lateral bundling [8], [10], [11]. Conversely, *E. coli* proteins MinC, SlmA and ClpXP destabilize FtsZ fibers and promote disassembly [7], [12]–[14]. Of these modulators of FtsZ assembly, several, including FtsA, ZipA, ClpXP and MinC, have been demonstrated to interact with a region of FtsZ near the C-terminus that contains a highly conserved sequence, referred to as the conserved core [7], [8], [15], [16]. MinC has been suggested to have a second interaction with FtsZ near the GTP-binding site at the interface between adjacent protomers [17]. MinC functions to prevent lateral bundling of FtsZ fibers and longitudinal polymer assembly [18].

Although it degrades FtsZ in vivo and in vitro, ClpXP is not an essential protein in *E. coli* for cell division or other cellular functions [19]. However, it modulates cell division by lowering the concentration of FtsZ, thereby shifting the dynamic equilibrium away from the polymerized form of FtsZ [7]. Several other modulators of FtsZ assembly are also not essential, including the FtsZ-associated proteins ZapA, ZapB, ZapC and ZapD [20]. Unlike the other modulators of FtsZ assembly, ClpXP is not a dedicated cell division protein and degrades many diverse protein substrates [21].

In the present study we report the identification of two regions near the FtsZ C-terminus that are important for degradation of FtsZ by ClpXP. One region is located 30 amino acids away from the C-terminus in an unstructured linker and the other region includes residues near the C-terminus. Our results suggest that ClpX recognizes FtsZ through dual contacts and residues in both regions are important for degradation by ClpXP. Although FtsZ degradation does not require an adaptor, degradation is inhibited when the SspB adaptor binding site on the ClpX N-domain is occupied by a peptide containing 10 C-terminal amino acid residues of SspB. We also demonstrate in vitro that FtsZ degradation is reduced in the presence of excess MinC, which is consistent with both MinC and ClpXP interacting with an overlapping region of FtsZ near the C-terminus.

## Experimental Procedures

### Bacterial strains and plasmids

*E. coli* strains and plasmids used in this study are listed in Table S1. Bacteria were grown in Lennox (LB) liquid broth at 30°C in the presence of ampicillin and arabinose, where indicated. The chromosomal *araE* promoter was replaced in MG1655 by P1 transduction with the constitutive promoter P_CP18_ to regulate expression of the high-capacity transporter (*araE*) [22], [23]. Plasmids encoding FtsZ mutant proteins were constructed by site-directed mutagenesis of pBAD-FtsZ, pGfp-FtsZ and pEt-FtsZ using the Quik-Site II Mutagenesis kit (Agilent) [7], [24], [25].

### Proteins and Peptides

*E. coli* FtsZ [7], ClpX [26], ClpP [27] and GFP-ssrA [28] proteins were expressed and purified as described previously. ClpX(E185Q) was purified like wild type ClpX [26]. The C-terminal SspB peptide, XB, with the sequence NH2-RGGRPALRVVK-COOH was purchased from Life Technologies. MinC was cloned into vector pET-24b (EMD-Millipore). Expression was induced in BL21(λDE3) cells (EMD-Millipore USA) (Table S1) at 30°C by adding 0.5 mM β-D-isopropyl-thiogalactoside after cells reached an O.D_600_ of 1.2. After 3 h of induction, cells were harvested by centrifugation at 6,000×*g* for 20 min, resuspended in 25 mM Tris-HCl, pH 8.0, 50 mM KCl, 10% glycerol, 1 mM EDTA and 1 mM TCEP [tris(2-carboxyethyl)phosphine], and then lysed by French press. The cell lysate was centrifuged at 35,000×*g* for 30 min at 4°C. MinC was purified from the soluble cell extract by chromatography on a Q sepharose column. Bound proteins were eluted with a KCl gradient (50–600 mM). Fractions containing MinC were fractionated on a sephacryl S-100 column equilibrated with 25 mM Tris-HCl, pH 7.5, 100 mM KCl, 10% glycerol and 1 mM TCEP. Protein concentrations are reported for ClpX hexamers, ClpP tetradecamers, MinC dimers and FtsZ monomers.

FtsZ mutant proteins were expressed in *E. coli* BL21 (λDE3) and purified like wild type FtsZ as described [7]. To incorporate fluorescent labels into active FtsZ wild type or mutant proteins, stable polymers were formed by adding GTP in the presence of CaCl_2_, then labeled with Alexa fluor 350, 488 or 670 succinimidyl ester (Life Technologies) to a degree of labeling ranging from 0.5–5.0 mol/mol as described [29]. Fluorescent FtsZ wild type and mutant proteins were depolymerized as described to obtain active labeled protein [30].

### Degradation Reactions

Fluorescent wild type or mutant FtsZ (10 µM) was incubated in assembly buffer [50 mM MES (morpholino-ethane-sulfonic acid), pH 6.5, 50 mM KCl and 10 mM MgCl_2_] with 25 µg/ml, acetate kinase and 15 mM acetyl phosphate. Where indicated, 2 mM GTP was added and reactions were incubated for 3 min at room temperature to promote FtsZ polymer formation. After polymerization, ClpX, ClpP and 4 mM ATP were added, where indicated, at the start of the degradation reaction. After incubation for 30 min, degradation reactions were stopped by the addition of 25 mM EDTA. Reactions were filtered on Nanosep ultrafiltration membranes (Pall Life Sciences) (MWCO 10 kDa), pre-washed with 100 mM NaCl containing 0.01% Triton X-100, by centrifugation at 16,000×*g* for 20 min. Total fluorescence of peptides in the eluent was measured using a Cary fluorometer. Background correction was made by subtracting the fluorescence of the eluting volume from reactions containing fluorescent FtsZ wild type or mutant protein, but without ClpXP.

Degradation reactions containing FtsZ in the presence of MinC were performed as above, except the final concentration of FtsZ present in the reaction was 5 µM. MinC was included in the FtsZ degradation reaction at final concentrations of 2, 5, 10 and 20 µM.

GFP-ssrA degradation was monitored by measuring the loss of fluorescence with time as described in reactions containing ClpX, ClpP, ATP and MinC, where indicated [28].

### Functional assays of FtsZ mutant proteins in vivo

*E. coli* strain MCZ84, containing the chromosomal *ftsZ84* gene, was transformed with arabinose inducible pBAD expression plasmids listed in Table S1 encoding FtsZ wild type and mutant proteins. Strains were grown overnight at 30°C in Lennox Broth containing ampicillin (100 µg ml^−1^). Stationary phase cultures were diluted 1∶100 in LB broth containing 0.05% NaCl, 0.02% arabinose and ampicillin, and grown at 42°C for 4 h. Colony forming units (CFU) were determined by dilution plating onto LB agar containing ampicillin, then growing the colonies overnight at the permissive temperature.

### Assembly Characterization of FtsZ mutant proteins

FtsZ wild type and mutant proteins were assayed for GTP hydrolysis using the phosphate detection reagent Biomol Green (Enzo Life Sciences). Reactions containing 5 µM FtsZ and 1 mM GTP in assembly buffer were incubated at 30°C for 15 min. The amount of free phosphate was measured in reactions at 0 and 15 min by comparison to a phosphate standard curve. Rates were calculated by measuring the amount of free phosphate released during the incubation period.

To measure polymerization by light scattering, wild type and mutant FtsZ polymers were assembled in reactions (80 µl) containing assembly buffer and 8 µM FtsZ. Polymerization was monitored with time after the addition of 1 mM GTP by light scattering using a Cary Eclipse fluorescence spectrophotometer with excitation and emission wavelengths set to 450 nm and 5 nm slit widths. Baseline readings were collected for two min, GTP was added and light scattering was measured for 30 min. FtsZ wild type and mutant polymers were visualized by negative staining with uranyl acetate and electron microscopy as described [48].

### Polymerization Assays with MinC

Fluorescent FtsZ polymers were assembled by mixing 5 µM fluorescence-labeled FtsZ in assembly buffer in the presence of 25 µg/ml acetate kinase and 15 mM acetyl phosphate. GTP (2 mM) was added and reactions were incubated for 3 min. Where indicated MinC, ClpX, ClpP and 4 mM ATP were added, and reactions were incubated for 10 min. FtsZ polymers were collected by centrifugation for 30 min at 129,000×*g* at 23°C. Supernatants and pellets were resuspended in assembly buffer containing 0.1 M NaCl and 0.005% Triton X-100. Total fluorescence was measured in supernatants and pellets.

### Co-sedimentation Assays

FtsZ polymers were assembled by adding 2 mM GTP to reactions containing assembly buffer, 25 µg/ml acetate kinase and 15 mM acetyl phosphate with 250 pmol FtsZ wild type or mutant protein. After incubating the reaction for 3 min to allow for GTP-dependent polymerization, 1.5, 5.0 or 12.5 pmol ClpX(E185Q) and 4 mM ATP were added to the reaction. The final reaction volume was 25 µl. After incubating for 10 min, reactions were centrifuged at 129,000×*g* for 30 min. Supernatants and pellets, resuspended in an equivalent volume of assembly buffer, were analyzed by SDS-PAGE and Coomassie staining. The relative amounts of ClpX(E185Q) in supernatant and pellet fractions were quantified by densitometry, and pmol of ClpX(E185Q) in the pellet fraction was calculated. Values were background-corrected by subtracting the total pmol of ClpX(E185Q) present in the pellet under identical conditions but omitting GTP and normalized to the amount of polymerized wild type or mutant FtsZ detected, which ranged from 99 to 174 pmol.

## Results

### The C-terminal region of FtsZ is important for recognition and degradation by ClpXP

**Figure 1:**
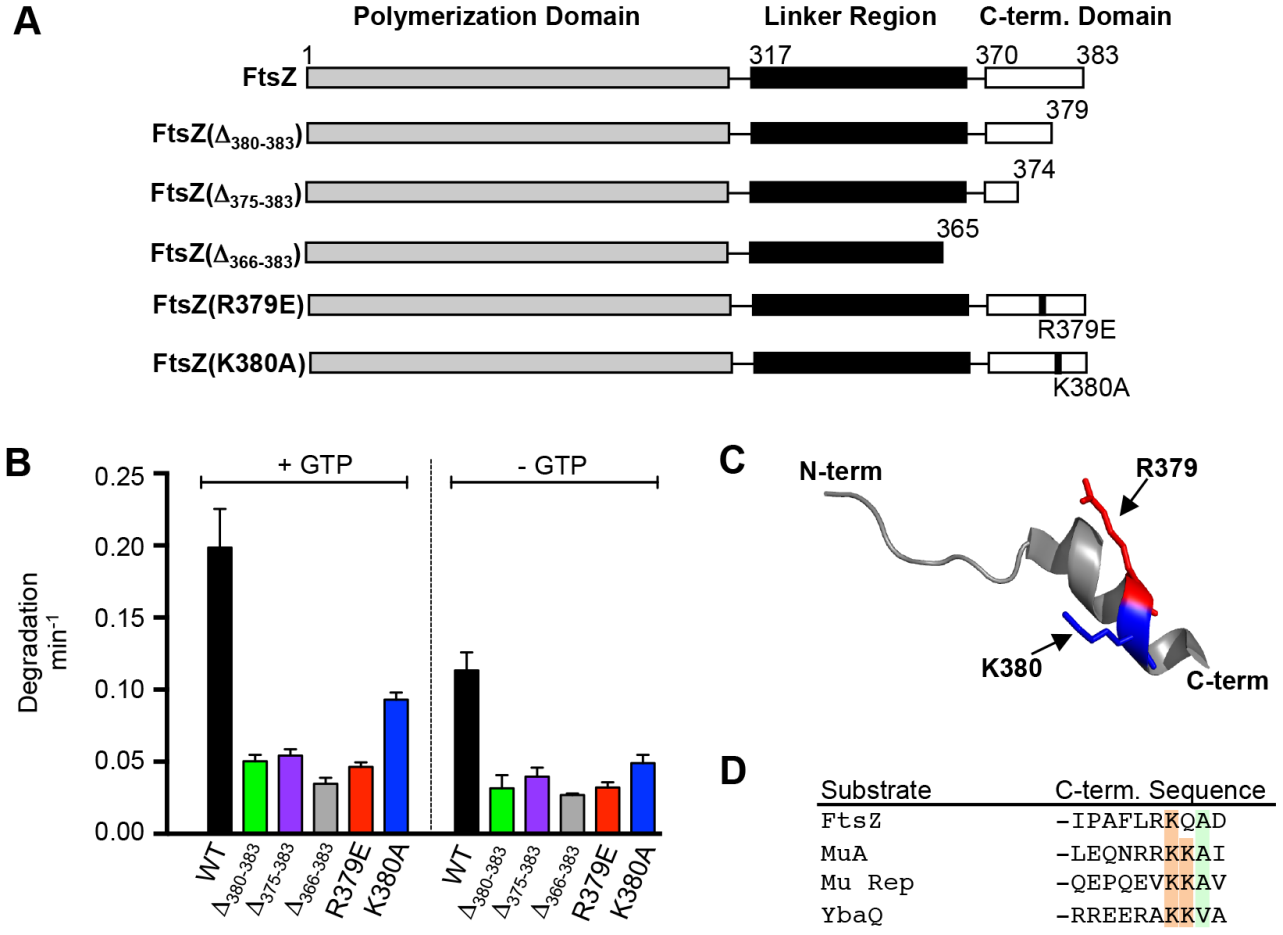
The FtsZ C-terminus is important for ClpXP degradation. A. Linear schematic diagram of FtsZ protein separated into three regions: the polymerization domain (amino acids 1 through 316), the unstructured linker (amino acids 317 through 369) and the C-terminal domain or conserved core region (amino acids 370 through 383). The C-terminal FtsZ deletions and substitution mutations used here are presented. B. Comparison of rates of degradation of FtsZ wild type and mutant proteins in the presence and absence of GTP from in vitro degradation reactions containing 10 µM wild type or mutant fluorescent FtsZ and 1 µM ClpXP. C. Structural model of the C-terminal alpha-helical region of FtsZ, residues 367 through 383, that cocrystallized with ZipA (PDB entry 1F47) [32]. Side chains are shown for R379 (red) and K380 (blue). D. Alignment of the C-terminal amino acid sequences of several proteins recognized by ClpX. C-terminal sequences shown belong to the consensus C motif-2 family of ClpX recognition tags (R/H-x-K/R-K-Φ with x representing any amino acid and Φ representing a hydrophobic amino acid residue) [21]. In B, data from 3 replicates are presented as mean ± SEM.

The region of FtsZ referred to as the “conserved C-terminal core”, which includes residues 370 through 379 [16], is located near the C-terminus (Fig. 1A). Residues 380 through 383 of FtsZ are less conserved and referred to as the C-terminal variable region [31]. The conserved core region contains residues that interact with several cell division proteins, including FtsA, ZipA and MinC [7], [8], [15], [16]. In addition residues within 18 amino acids of the C-terminus, 366 to 383, are important for degradation by ClpXP [7]. To elucidate the residues within this region of FtsZ that are important for ClpX recognition we constructed FtsZ deletion and substitution mutants (Fig. 1A). FtsZ(Δ_380-383_) is a deletion mutant that lacks the C-terminal four amino acid variable region and FtsZ(Δ_375-383_) is a deletion mutant lacking the C-terminal nine amino acids. We also engineered substitution mutations at two positively charged residues near the C-terminus, R379E and K380A. We determined the rates of degradation of fluorescent wild type and mutant FtsZ by ClpXP by monitoring the appearance of degradation products using an ultrafiltration assay. As we previously observed, FtsZ was degraded at an approximately 2-fold faster rate in the presence of GTP, the condition that promotes FtsZ polymerization, than in the absence of GTP (0.20 min^−1^ and 0.11 min^−1^ in the presence and absence of GTP, respectively) (Fig. 1B). However, FtsZ mutant proteins lacking either four or nine residues from the FtsZ C-terminus were degraded at ∼75% reduced rates compared to wild type FtsZ in the presence of GTP and ∼65% reduced rates compared to wild type FtsZ in the absence of GTP. The reduction in the degradation rates of FtsZ(Δ_380-383_) and FtsZ(Δ_375-383_) were similar to the reduction in the degradation rate previously observed for FtsZ(Δ_366-383_) (Fig. 1B) [7]. We also observed that FtsZ substitution mutants FtsZ(R379E) and FtsZ(K380A) were degraded by ClpXP at ∼75% and ∼55% reduced rates, respectively, compared to wild type FtsZ in both the presence and absence of GTP (Fig. 1B). These results indicate that the positively charged residues R379 and K380 of FtsZ are important for degradation of FtsZ by ClpXP. Taken together, our results suggest that ClpX recognizes FtsZ through a C-terminal recognition motif and further implicates amino acid residues R379 and K380 as important for degradation by ClpXP.

Structural models of an *E. coli* FtsZ C-terminal peptide containing residues 367 through 383, which cocrystallized with the FtsZ-binding domain of ZipA, show a nine amino acid linear alpha helix at the FtsZ C-terminus (Fig. 1C) [32]. Amino acid residues R379 and K380 are located within the C-terminal helix, and the side chains extend outward. Cocrystallization of this region with FtsA from *Thermatoga maritima* showed an alternative configuration, with the helix containing a 90 degree bend, which could suggest that the FtsZ tail may be capable of adopting different conformations [33]. In the absence of structural information concerning the configuration of the *E. coli* FtsZ C-terminal tail with ClpX, this region has been illustrated based on the *E. coli* model with ZipA (Fig. 1C) [32].

Our results indicate that the FtsZ C-terminal region, containing positively charged amino acid residues R379 and K380, is important for degradation of FtsZ by ClpXP. ClpX is known to recognize the C-termini of substrates that contain one of two distinct recognition motifs; one motif, referred to as C motif-1, resembles the C-terminal LAA residues of the ssrA tag sequence, and a second motif, referred to as C motif-2, resembles the C-terminal sequence of the degradation substrate from bacteriophage Mu, MuA, a DNA transposase [21]. An alignment of the FtsZ C-terminus with the C-terminal 10 amino acids from substrates that contain recognition tags bearing similarity to the C motif-2 consensus motif (R/H-x-K/R-K-Φ with x representing any amino acid and Φ representing a hydrophobic amino acid residue) is shown in Fig. 1D [21]. The alignment shows similarities between the C-terminus of FtsZ and other ClpXP degradation substrates, including K380 and the nearby hydrophobic amino acid A382. The reduced rate of degradation observed for the mutant protein FtsZ(Δ_380-383_), which is missing both K380 and A382, compared to wild type FtsZ is consistent with the suggestion that these residues are important for degradation by ClpXP.

Additional substitution mutations in the conserved alpha-helical region near the FtsZ C-terminus were constructed and tested for degradation by ClpXP (Fig. S1). We observed ∼50% reduced rates of degradation of two mutant proteins FtsZ(F377A) and FtsZ(P375G) in the presence of GTP, compared to wild type FtsZ; however, we did not observe similar reductions when GTP was omitted (Fig. S1). One mutant protein, FtsZ(L378A), was degraded at a 1.9-fold faster rate than wild type FtsZ in the absence of GTP, but not in the presence of GTP. Together, these results suggest that amino acid residues in the vicinity of the recognition motif may modulate the rate of degradation in the presence or absence of GTP.

### The linker region of FtsZ also contains residues important for degradation by ClpXP

**Figure 2:**
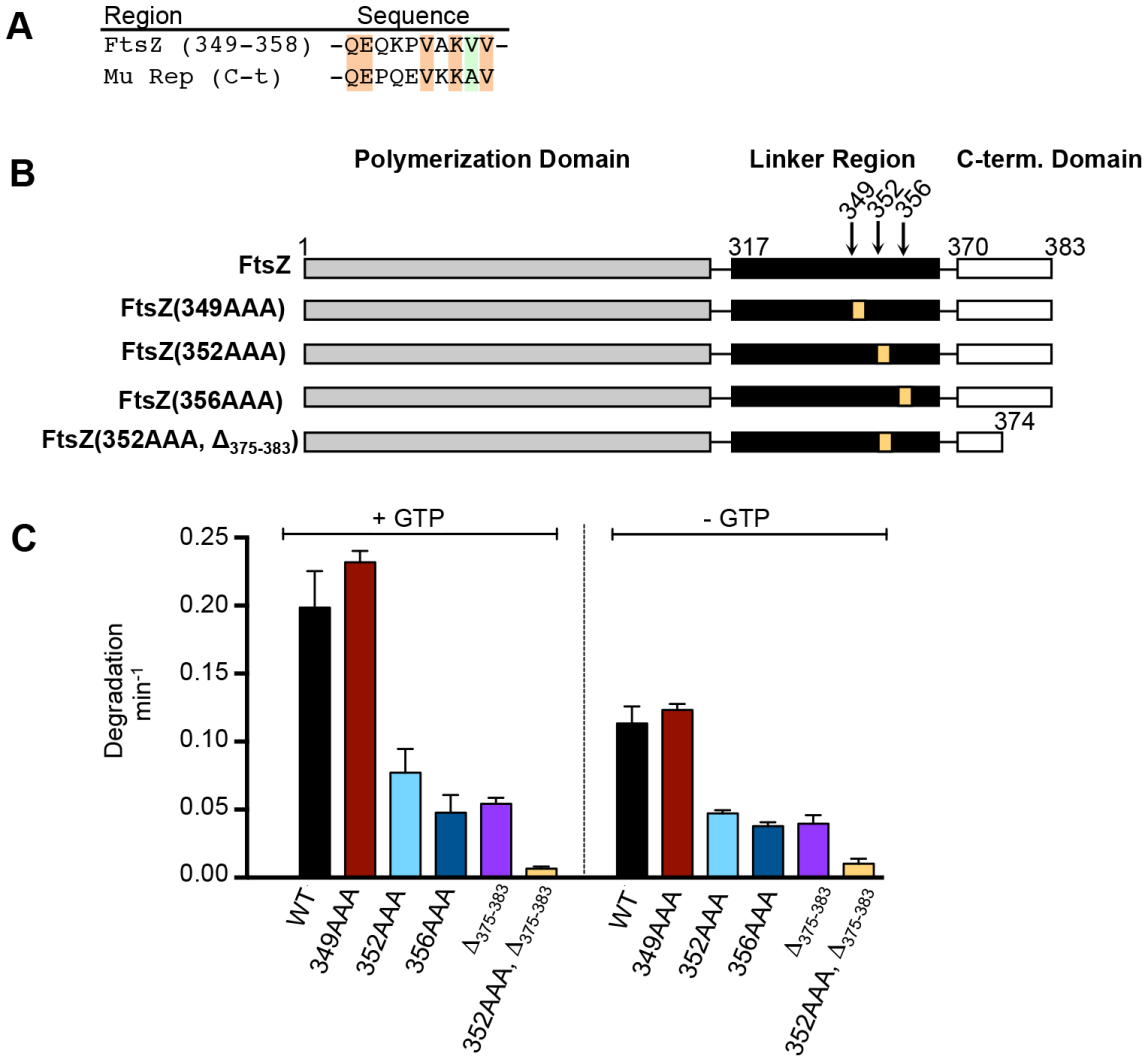
Residues in the linker region of FtsZ are important for degradation by ClpXP. A. Alignment of FtsZ residues 349 through 358 from the linker domain with the C-terminal ClpX recognition region of Mu repressor protein from phage Mu. B. Linear schematic diagram of FtsZ showing regions of the linker that were tested by triple alanine scanning mutagenesis of wild type FtsZ and truncated FtsZ(Δ_375-383_). C. Comparison of rates of degradation of wild type and mutant FtsZ, proteins in the presence and absence of GTP from in vitro degradation reactions containing 10 µM wild type or mutant fluorescent FtsZ and 1 µM ClpXP. Data from 3 replicates are presented as mean ± SEM.

Our observation that deletion of 4, 9, or 18 residues from the FtsZ C-terminus reduced degradation by ClpXP but did not abolish it (Fig. 1B), suggests that additional residues in FtsZ may also participate in a ClpX interaction. Therefore we examined the linker region of FtsZ, residues 317 through 369, between the polymerization domain and the conserved C-terminal domain for an additional site of interaction for ClpX (Fig. 2A). The linker region overall is poorly conserved in bacteria, and structure prediction algorithms suggest that the linker has little secondary structure. Erickson and colleagues recently showed that the linker region is an intrinsically disordered peptide and because it could be replaced with almost any sequence of similar length, it likely functions as a bridge, linking the FtsZ polymerization domain to the C-terminal protein interaction sites via a flexible tether [34]. By sequence examination, we identified a 10-residue motif in the linker region (349 QEQKPVAKVV 358) that contains 60% homology to the ClpXP recognition signal in Mu repressor, however the region did not strictly adhere to the C motif-2 consensus motif (R/H-x-K/R-K-Φ) (Fig. 2A). To probe this region further and test if residues in this potential site are also important for the interaction with ClpX, we constructed several triple alanine substitutions in the region, shown in Fig. 2B. We compared rates of degradation of the FtsZ linker mutant proteins to wild type FtsZ in the presence and absence of GTP (Fig. 2C). We observed that two of the mutant proteins we constructed, FtsZ(352AAA) and FtsZ(356AAA), were degraded at 60–75% slower rates than wild type FtsZ in both the presence and absence of GTP. However, a nearby triple alanine substitution mutant, 349AAA, was degraded at a rate similar as wild type FtsZ (Fig. 2C).

To test if the two regions we identified are both important for degradation, we constructed FtsZ(352AAA, Δ_375-383_). We observed that mutation of both sites in FtsZ, one in the linker and the other near the C-terminus, abolished degradation by ClpXP in the presence or absence of GTP (Fig. 2C). Taken together, our data demonstrate that two regions of FtsZ separated by approximately 20 residues promote the recognition and degradation of FtsZ. Disruption of both regions in FtsZ prevents degradation by ClpXP.

### FtsZ mutant proteins hydrolyze GTP and exhibit GTP-dependent polymerization

**Figure 3:**
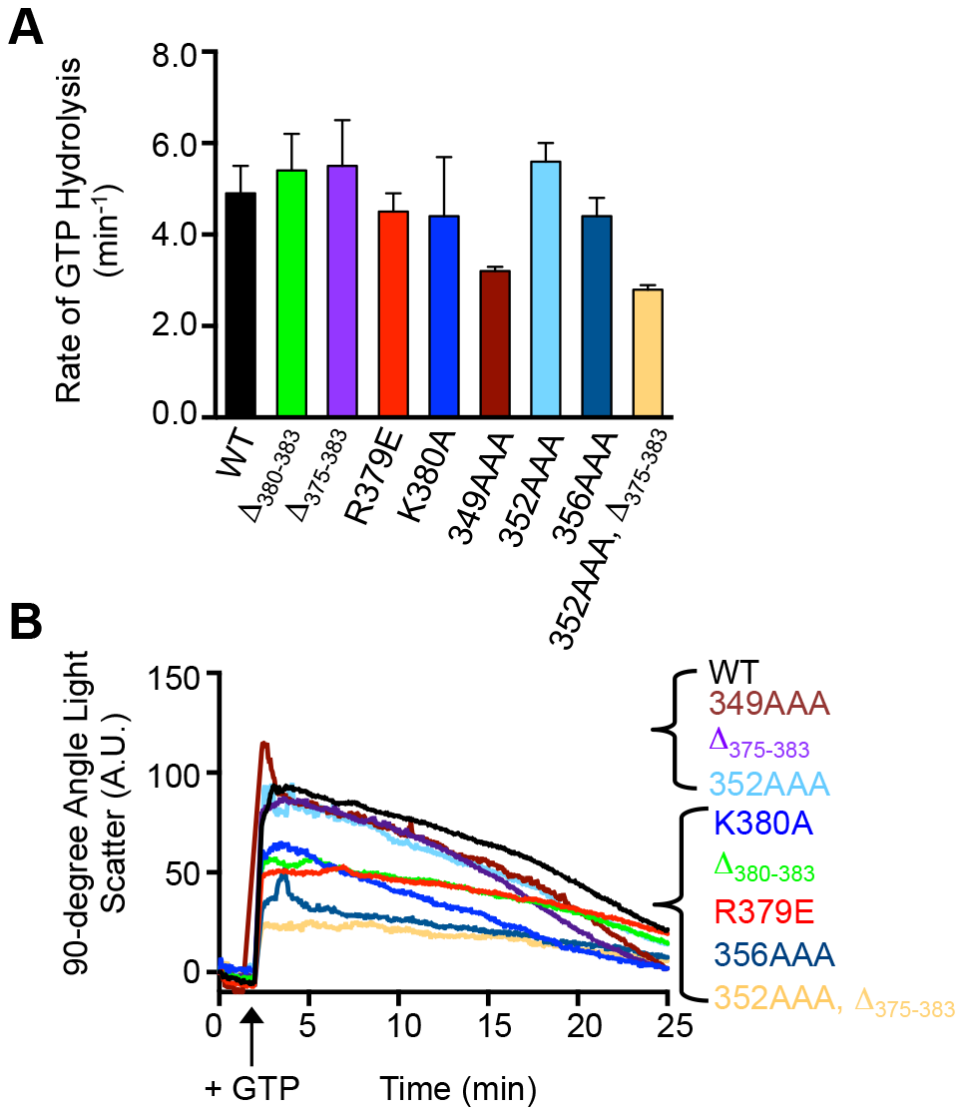
FtsZ mutant proteins with C-terminal mutations hydrolyze GTP and assemble into polymers. (A) Rates of GTP hydrolysis were measured in reactions containing FtsZ wild type or mutant (5 µM) and GTP (1 mM) as described in *Experimental Procedures*. Data from 3 replicates are presented as mean ± SEM. (B) GTP-dependent assembly of FtsZ wild type and mutant proteins (8 µM) was monitored by 90° light scattering as described in *Experimental Procedures*. A baseline was collected for 2 min, then GTP was added when indicated, to stimulate polymerization. Light scattering was measured for 25 min. Data shown is representative of 3 replicates.

To test if FtsZ mutant proteins are functional for GTP-dependent assembly and GTP hydrolysis, we performed a characterization of FtsZ mutant proteins in vitro. As expected, all of the mutant proteins hydrolyze GTP. Many of the FtsZ mutant proteins exhibit rates of GTP hydrolysis similar to wild type FtsZ (4.9±0.6 min^−1^), however we observed a ∼40% slower rate of GTP hydrolysis by FtsZ(349AAA) and FtsZ(352AAA, Δ_375-383_) compared to wild type FtsZ (Fig. 3A). In addition all of the mutant proteins showed an increase in light scatter when GTP was added indicating that they polymerize in a GTP-dependent manner (Fig. 3B). However, the increase in light scatter upon addition of GTP was ∼30–70% less with FtsZ(R379E), FtsZ(K380A), FtsZ (Δ_380-383_), FtsZ(356AAA) and FtsZ(352AAA, Δ_375-383_) compared to wild type FtsZ. Visualization by electron microscopy and negative staining showed that all of the FtsZ mutant proteins tested formed single-stranded filaments in the presence of GTP (Fig. S2), including FtsZ(352AAA, Δ_375-383_) which showed reduced GTPase activity and GTP-stimulated light scatter. We also observed fiber bundles in addition to single stranded filaments by electron microscopy for mutant proteins FtsZ(R379E), FtsZ(352AAA) and FtsZ(352AAA, Δ_375-383_).

### A region in the ClpX N-domain overlapping the SspB binding site is important for FtsZ recognition and degradation by ClpXP

**Figure 4:**
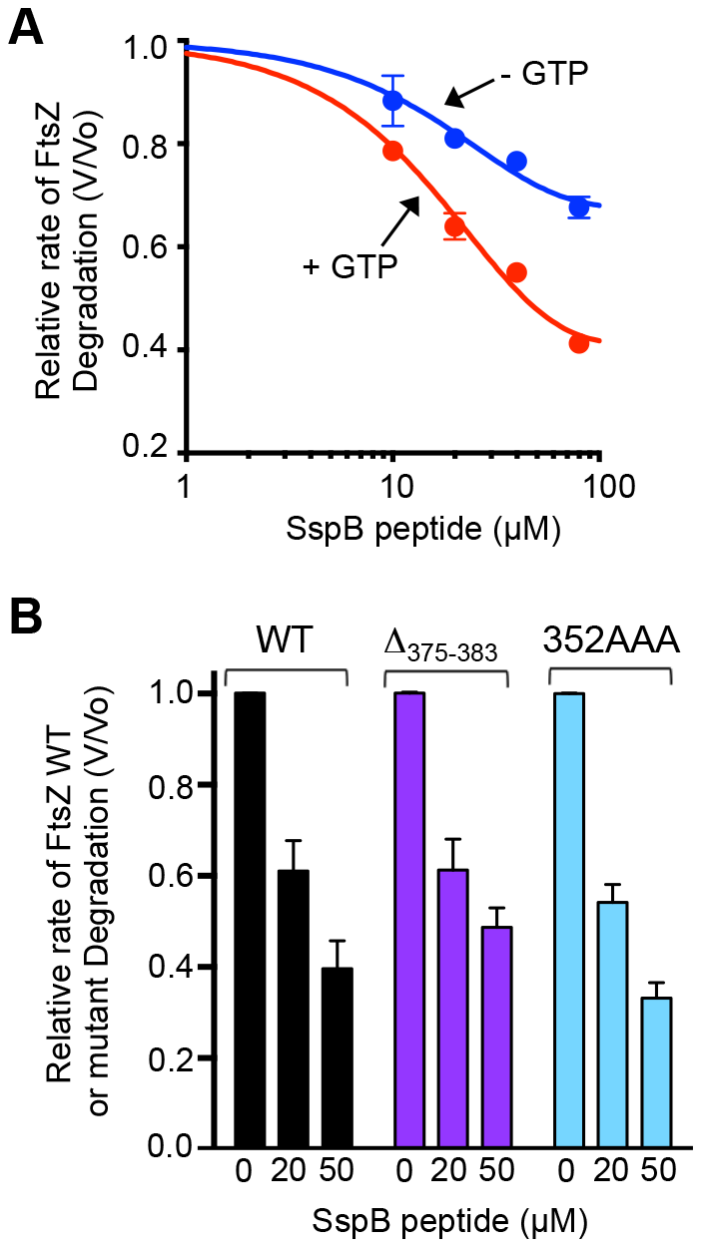
A peptide corresponding to the C-terminus of the SspB adaptor inhibits FtsZ degradation. A. Relative rate of FtsZ degradation by ClpXP in the presence and absence of GTP with increasing concentration of SspB peptide (10, 20, 40 or 80 µM). Relative rate of FtsZ degradation was defined by V/V_o_, where V is equal to the rate in the presence of SspB peptide and V_o_ is equal the rate in the absence of SspB peptide. Degradation reactions contained 15 µM fluorescent FtsZ, 0.75 µM ClpXP, ATP and, where indicated, GTP. Data were fit to a nonlinear dose-response inhibitor curve. B. Comparison of relative rates of degradation of wild type FtsZ, FtsZ(Δ_375-383_) and FtsZ(352AAA) in the presence of 0, 20 or 50 µM SspB peptide. Reactions contained 10 µM FtsZ wild type or mutant protein, 0.75 µM ClpXP with GTP and ATP. In A and B data from 3 replicates are presented as mean ± SEM.

We next wanted to investigate the region of ClpX N-domain that interacts with FtsZ. The N-domain of ClpX, comprised of residues 1 through 61, is important for recognition and degradation of FtsZ [7]. The ClpX N-domain is also essential for recognition and degradation of lambda O protein and adaptor-mediated substrate recognition and degradation, including ssrA-tagged proteins in the presence of adaptor protein SspB [3], [6], [35]. A peptide corresponding to the 10 C-terminal amino acids of SspB, referred to as the XB peptide, binds directly to the ClpX N-domain and inhibits SspB-stimulated degradation of ssrA tagged substrates [36], [37]. The XB peptide also inhibits degradation of lambda O protein, and structural studies suggest that the lambda O binding site on ClpX overlaps with the SspB binding site [3], [35]. Since the ClpX N-domain is essential for recognition and degradation of FtsZ by ClpXP [7], we tested if degradation is susceptible to inhibition by the XB peptide. We monitored degradation of FtsZ in the presence of increasing amounts of XB peptide with and without GTP. We observed that addition of XB peptide inhibited degradation of FtsZ in the presence and absence of GTP in a concentration-dependent manner (Fig. 4A). The XB peptide is approximately two-fold more inhibitory against FtsZ degradation in the presence of GTP than in the absence of GTP. These results show that the region of SspB that interacts with the N-domain of ClpX is an inhibitor of FtsZ degradation. They suggest that the FtsZ binding site on ClpX overlaps with the SspB adaptor-binding site. These results also demonstrate that FtsZ polymers are more susceptible to inhibition by the peptide than FtsZ monomers.

Next we tested if both regions of FtsZ that promote ClpXP degradation, the linker and the C-terminal region, are susceptible to peptide inhibition by monitoring degradation of FtsZ(352AAA) and FtsZ(Δ_375-383_) in the presence and absence of the XB peptide. Our results show that degradation of FtsZ(352AAA) and FtsZ(Δ_375-383_) is inhibited by the XB peptide to a similar extent as wild type FtsZ (Fig. 4B), suggesting that the SspB binding region of the ClpX N-domain is important for interaction with both of the FtsZ degradation signals, the one in the linker and the one near the C-terminus.

### Association of ClpX with wild type and mutant FtsZ polymers

**Figure 5:**
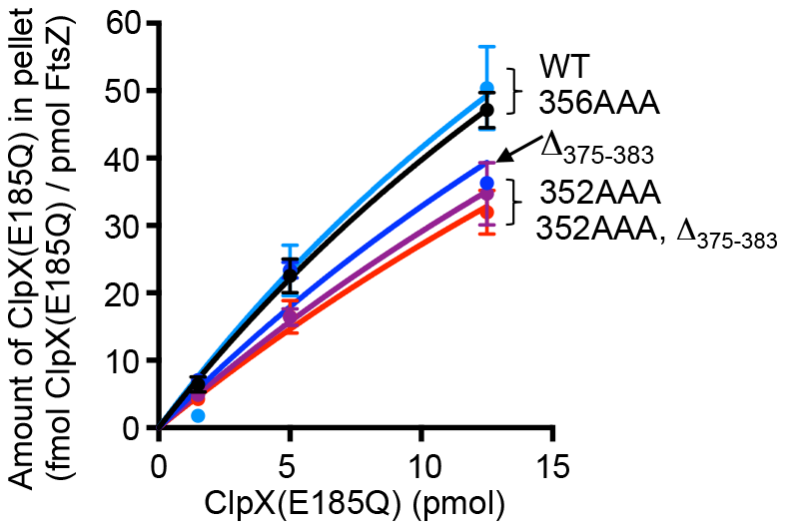
Association of FtsZ wild type and mutant polymers with ClpX. The interaction between ClpX and FtsZ was measured by monitoring the fraction of ClpX ATP hydrolysis mutant, ClpX(E185Q), that co-pellets with FtsZ wild type or mutant protein in the presence of GTP. Pelleted ClpX(E185Q) and FtsZ was quantified by Coomassie staining of SDS-PAGE gels and densitometry. Data from at least 3 replicates are presented as mean ± SEM.

Having shown that FtsZ degradation by ClpXP involves two regions of FtsZ (Fig. 1B and 2C), we wanted to determine if the FtsZ mutant proteins that are poorly degraded by ClpXP are defective in an interaction with ClpX. To test this, we performed a co-pelleting assay to monitor the association of polymerized FtsZ wild type or mutant protein with an ATP hydrolysis defective mutant of ClpX, ClpX(E185Q), which can form stable interactions with substrates in the presence of ATP [38]. We observed that ClpX(E185Q) co-pelleted with wild type FtsZ polymers in a concentration-dependent manner (Fig. 5). ClpX(E185Q) also associated with FtsZ(356AAA) polymers. However, slightly less ClpX(E185Q) co-pelleted with polymers containing FtsZ(Δ_375-383_), FtsZ(352AAA) or FtsZ(352AAA, Δ_375-383_) than wild type FtsZ, suggesting that these mutants are slightly defective for the association with ClpX(E185Q).

### ClpXP and MinC compete for FtsZ in vitro

**Figure 6:**
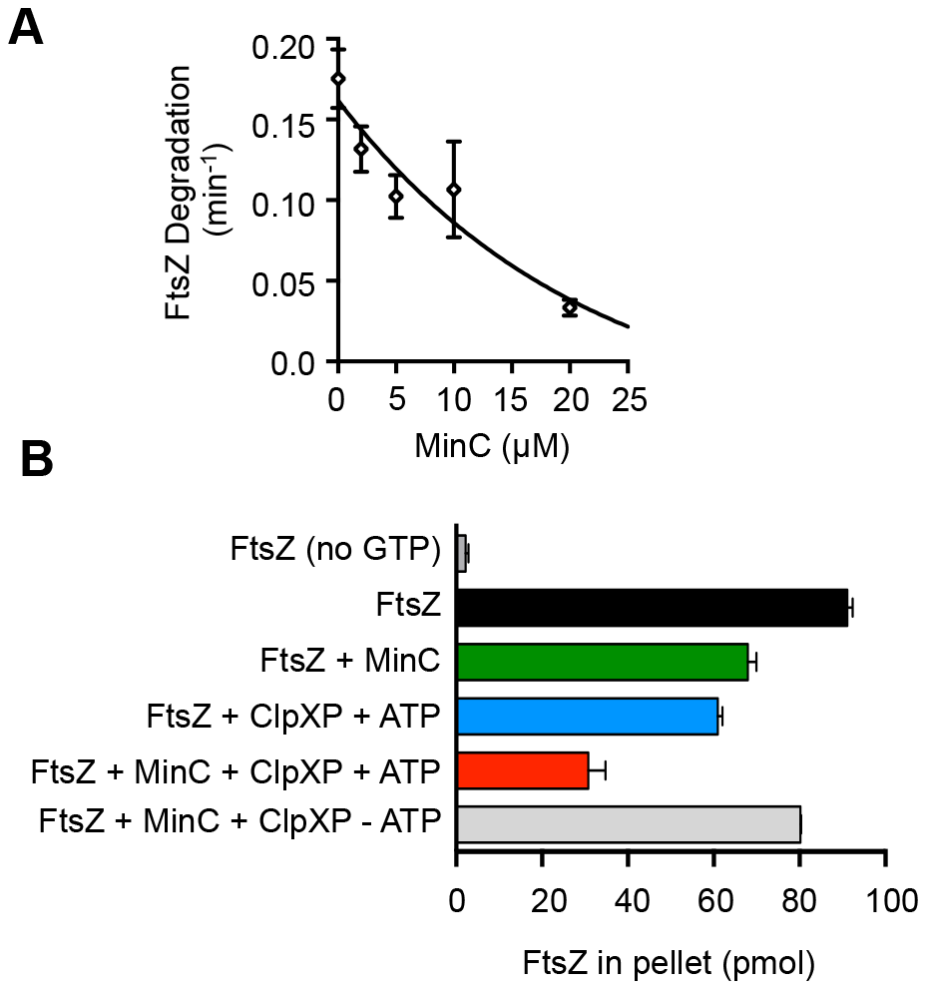
MinC competes with ClpXP for FtsZ in vitro. A. MinC was included in degradation reactions containing FtsZ with GTP and ClpXP (0.5 µM). B. FtsZ (5 µM; 125 pmol/reaction) was first preincubated with GTP (2 mM) and then incubated with MinC (2 µM), ClpXP (0.5 µM) and ATP, as indicated. FtsZ polymer disruption was monitored by measuring the amount of fluorescent FtsZ present in high-speed centrifugation pellets. In A and B data from 3 replicates are presented as mean ± SEM.

MinC interacts with FtsZ in the C-terminal domain and isoleucine 374 has been shown to be important for this interaction [15]. Therefore if ClpX associates with FtsZ near the C-terminus, an interaction with MinC could potentially mask residues important for recognition by ClpX and prevent degradation in vitro. To test if MinC competes with ClpXP for FtsZ in vitro, we monitored degradation of fluorescent FtsZ by ClpXP in the presence of increasing amounts of MinC. We observed that MinC inhibited FtsZ degradation with 80% inhibition resulting from a four-fold excess of MinC dimer over FtsZ monomer (Fig. 6A). In a control experiment, we observed that degradation of GFP-ssrA by ClpXP was not inhibited by MinC (Fig. S3). One interpretation of our results is that there is competition between ClpXP and MinC in vitro for binding the C-terminal domain of FtsZ.

Both MinC and ClpXP have independently been shown to destabilize FtsZ polymers in vitro leading to polymer disassembly [7], [18]. In our in vitro competition experiment, we observed inhibition of FtsZ degradation when MinC was in excess over FtsZ. However, in vivo FtsZ is in large excess over MinC, based on estimates of 10,000 and 400 molecules of FtsZ and MinC, respectively, per cell [39], [40]. Consequently, multiple modulators of FtsZ assembly are likely acting independently and at the same time on the large amount of FtsZ inside the cell. Therefore, to test if MinC and ClpXP act concurrently to disrupt FtsZ polymers in vitro when MinC and ClpXP are under limiting conditions, we monitored FtsZ polymer abundance after incubation with ClpXP and MinC. MinC was added first to preassembled FtsZ polymers, then ClpXP and ATP were added to the reaction. After a short incubation, the remaining FtsZ polymers were collected by centrifugation and quantified. The supernatant fractions, which were not quantified, contained mixtures of FtsZ monomers and dimers, as well as products of the degradation reaction when ClpXP and ATP were included. In control experiments, the addition of either ClpXP or MinC to FtsZ polymers caused a reduction in FtsZ polymer abundance by 33% for ClpXP and 25% for MinC (Fig. 6B). When ClpXP and MinC were both included in the reaction, there was a 65% reduction of FtsZ polymers. The reduction was similar to the additive value of the individual contributions of ClpXP and MinC, or approximately 60%, and dependent on the presence of ATP. These results suggest that under our conditions, MinC and ClpXP perform independent and concurrent disruption activities on FtsZ polymers in vitro.

### FtsZ C-terminal mutants are defective for function in vivo

**Figure 7:**
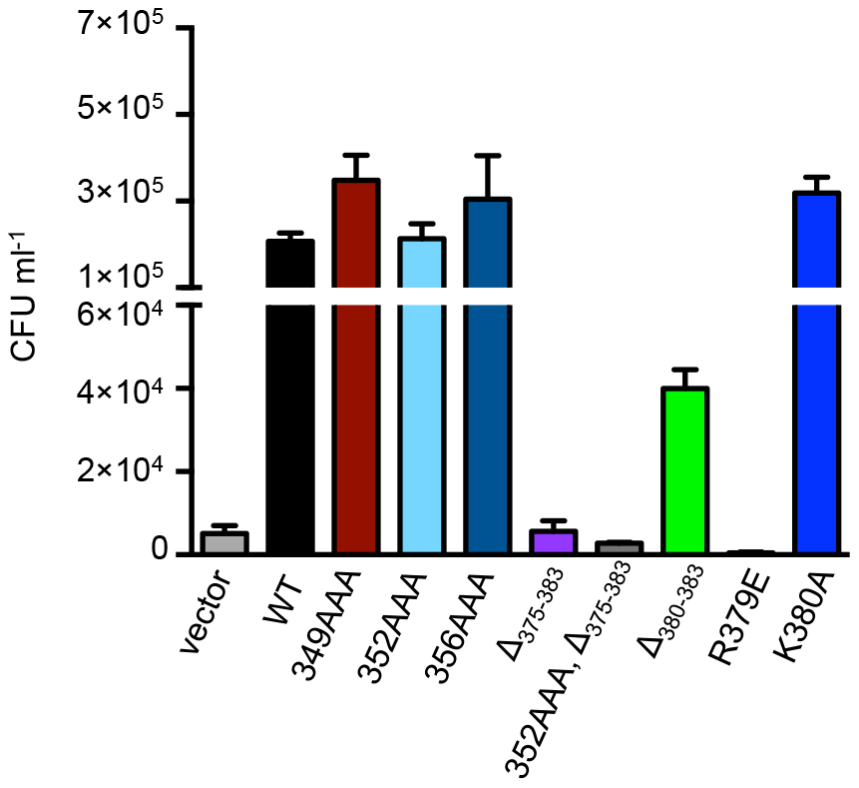
Mutations in the FtsZ C-terminal domain impair FtsZ function in vivo. FtsZ mutant proteins were tested for function in vivo by comparing CFUs of *ftsZ84* cells expressing FtsZ mutant proteins after incubation in liquid culture at the restrictive temperature (42°C) for 4 hours as described in *Experimental Procedures*. Data from 3 replicates are presented as mean ± SEM.

In *E. coli, clpX* and *clpP* are dispensable for growth and genetic deletion of *clpX* or *clpP* is not associated with division defects [7], [19]. In addition ClpXP-dependent phenotypes have not been described. Since the C-terminal region of FtsZ is essential for interactions with known cell division proteins as well as ClpXP, it is likely that any growth defects exhibited by our FtsZ mutant proteins are caused by failure of FtsZ to interact with essential cell division proteins including MinC, FtsA or ZipA. However, we tested FtsZ mutant proteins in vivo to determine if mutations in regions of FtsZ important for ClpXP degradation are also associated with defects in FtsZ function. We expressed each mutant FtsZ protein from a plasmid in an *ftsZ84* temperature sensitive strain [41] and measured the number of CFUs after incubation at 42°C. In control experiments, expression of wild type FtsZ from a plasmid supported growth of *ftsZ84* cells at 42°C, while cultures of cells carrying the control vector were unable to grow (Fig. 7). Expression of FtsZ(Δ_375-383_) and FtsZ(R379E) did not support growth and cells expressing FtsZ(Δ_380-383_) were partially functional, as expected since residues in this region are important for interactions with both ZipA and FtsA. Cells expressing FtsZ(K380A) grew slightly better than those expressing wild type FtsZ, while substitution mutations in residues 375 through 378 impaired FtsZ function in vivo (Fig. S4A), as previously reported [16]. Expression of the FtsZ linker mutant proteins FtsZ(349AAA), FtsZ(352AAA) and FtsZ(356AAA) support growth of *ftsZ84* cells at 42°C, similar to cells expressing wild type FtsZ (Fig. 7). However, cells expressing the double mutant FtsZ(352AAA, Δ_375-383_) were unable to grow. The cell growth defects detected in this assay likely reflect changes in interactions with essential cell division proteins such as MinC, FtsA or ZipA. In addition, no defects were detected in our assay in cells expressing FtsZ(352AAA) or FtsZ(356AAA), which are defective for degradation by ClpXP in vitro.

Next, we tested whether the FtsZ mutant proteins localize to Z-rings in wild type cells by expressing each FtsZ mutant protein as a green fluorescent protein (GFP) fusion from a plasmid. All GFP-FtsZ mutant proteins tested assembled into ring-like structures in dividing cells (Fig. S5). Expression of GFP-FtsZ proteins containing triple alanine substitutions in the linker region showed normal Z-rings and no obvious cell division defects. In contrast, many of the C-terminal mutations, including R379E and Δ_375-383_, were associated with mild cell filamentation and multiple Z-rings in some cells (Fig. S5 and Fig. S4B). Filamentation and Z-ring localization was observed in cells that expressed GFP-FtsZ proteins containing mutations at residues 375, 376, 377 and 378 (Fig. S4B) [16]. Cells expressing GFP-FtsZ(Δ_380-383_) or GFP-FtsZ(K380A) exhibited normal Z-ring formation and were not filamentous.

## Discussion

In this study, we have identified two regions near the FtsZ C-terminus that are important for proteolysis by ClpXP. The C-terminal end of FtsZ harbors a motif that resembles the C motif-2 family of ClpX recognition motifs proposed in Flynn, et al [21]. Like the ClpXP substrate MuA, the C-terminus of FtsZ contains positively charged residues, R379 and K380, which are important for degradation by ClpXP [4]. Our results further show that there is a second site important for degradation by ClpXP that is located 30 residues from the C-terminus of FtsZ in a highly flexible and unstructured linker region [34]. Another ClpXP substrate, MuA, also utilizes additional contacts to stabilize the ClpX-interaction, and the presence of multiple ClpX recognition sites in FtsZ may serve a similar function [4]. In FtsZ, both sites can function independently since both FtsZ(352AAA) and FtsZ(Δ_375-383_) are partially defective and inhibited by the SspB C-terminal peptide (Fig. 1B, 2C and 4B). A change in the rate of degradation could result from impaired recognition, unfolding, translocation into ClpP, or proteolytic cleavage. Co-pelleting assays between FtsZ mutants and an ATP hydrolysis-defective mutant of ClpX (Fig. 5) are consistent with the suggestion that the reduced rates of degradation may be due to defective recognition. However in our assay FtsZ(352AAA, Δ_375-383_) was only partially defective for an interaction with ClpX(E185Q), which could suggest that substrate engagement is impaired.

GTP-dependent polymerization of FtsZ shown here, and previously with the GTP-analog GMPCPP [7], enhances the rate of degradation of FtsZ (Fig. 1B), suggesting that the GTP-bound or polymer form of FtsZ may be recognized more efficiently. This could be due to exposure of additional contacts upon GTP-binding or polymerization, or due to enhanced association supported by multivalent interactions with polymerized FtsZ. Interestingly, C-terminal substitution mutants of FtsZ at residues P375, A376, or F377 are not stimulated for degradation in the presence of GTP compared to the absence of GTP (Fig. S1), suggesting that these residues could be important for stabilizing interactions with ClpX; however, GTP enhances the rate of degradation of FtsZ(Δ_375-383_), which does not contain residues 375 through 377, by ∼40% (Fig. 1). Multimerization may be important for enhancing degradation of low affinity substrates by ClpXP by increasing the local concentration of recognition sites and therefore promoting an interaction with ClpX. In our studies with FtsZ, we observed that the rate of degradation is maximal when both the C-terminal and linker regions are present and FtsZ is incubated under conditions that promote polymerization (Figs. 1B and 2C). Moreover, additional interactions between FtsZ and ClpX, which contains six N-domains per hexamer, may stabilize the interaction or function as a tether to promote additional recognition events.

We observed that FtsZ(R379E), which is poorly degraded by ClpXP in vitro, causes a severe growth defect (Fig. 7 and S5). Since there are no cell growth phenotypes associated with deletion of *clpX* or *clpP* in wild type *E. coli*, this result suggests that this residue is important for interactions with cell division proteins, such as MinC or FtsA, in addition to ClpX. Other substitution and deletion mutations introduced in the C-terminal conserved region of FtsZ also caused severe functional defects when expressed in *ftsZ84* cells and when expressed as GFP fusion proteins (Fig. 7, S4 and S5). In contrast, expression of FtsZ linker mutants FtsZ(352AAA) and FtsZ(356AAA), which are partially defective for degradation by ClpXP in vitro, in *ftsZ84* cells grown at the restrictive temperature did not result in obvious functional defects (Fig. 7). A recent study also reported no defects associated with the replacement of residues in the linker region [34].

The C-terminal variable region of FtsZ, which contains the last six residues of FtsZ, from *B. subtilis* has been shown to promote the lateral bundling of FtsZ polymers [31]. In contrast to the results with *B. subtilis* FtsZ, neither the variable region nor the unstructured linker region of *E. coli* FtsZ has been shown to promote bundling of filaments [31], [34]. The FtsZ C-terminal mutant proteins examined in our study form single-stranded polymers, however we observed several pairs or bundles of filaments by electron microscopy (Fig. S2), but they were not the dominant species observed.

Several proteins in the cell, both essential and non-essential, influence the dynamic assembly and disassembly of FtsZ. The major inhibitor of FtsZ polymerization in the bacterial cell is MinC. Our data demonstrate that like MinC, ClpXP is also capable of promoting disruption of FtsZ polymers (Fig. 6B). MinC binds to FtsZ and prevents assembly at the subunit interface and lateral interactions between FtsZ polymers [17], [18], [42]. Although it has been reported that ClpX can inhibit FtsZ polymerization in vitro under certain conditions, our previously published results indicate that FtsZ polymer disassembly activity requires ATP-dependent degradation [7], [43]. When MinC and ClpXP are in limiting concentrations compared to FtsZ and not in competition, then they function simultaneously to promote polymer disassembly (Fig. 6B). However, we observed that degradation of FtsZ by ClpXP is reduced by 80% in the presence of excess MinC, suggesting that MinC and ClpX are in competition and recognize overlapping regions of the FtsZ C-terminus. Recently, it was also reported that ZipA can similarly protect FtsZ from degradation by ClpXP, likely through preventing access to the FtsZ C-terminus [44]. Degradation of FtsZ by ClpXP occurs more efficiently under conditions that promote FtsZ polymerization (Fig. 1B) [7]. Therefore disassembly of FtsZ polymers by MinC could also contribute to the reduced FtsZ degradation observed in vitro in the presence of MinC.

FtsZ was initially identified as a ClpXP substrate in *E. coli* in a proteomics study to isolate kinetically-trapped ClpP complexes from cell lysates [21]. Recently, a similar proteomics study in *Staphylococcus aureus*, also identified FtsZ as a substrate for ClpP-mediated degradation [45]. Direct interactions between ClpX and FtsZ have also been reported in *Bacillus subtilis* and *Mycobacterium tuberculosis*, although FtsZ does not appear to be degraded by ClpXP in these organisms [46], [47]. In *E. coli*, the physiological effect of inhibiting FtsZ degradation in a cell where division is partially defective, such as the *ftsZ84* strain, is beneficial, resulting in partial suppression of the phenotype when *clpX* or *clpP* is deleted [25]. The role of FtsZ degradation during the division process in a wild type cell is less clear, however modest overexpression of ClpXP enhances FtsZ degradation and causes cellular filamentation associated with defective division [7]. Our results demonstrate that the specific degradation of FtsZ by ClpXP occurs through a complex recognition mechanism that is modulated by FtsZ conformation and may be impacted by the presence of other cell division proteins, including MinC and ZipA, and thus provides a mechanism for the cell to modulate division through proteolysis of FtsZ.

## Supporting Information

Figure S1 **Substitution of residues near the FtsZ C-terminus modulates the rate of degradation by ClpXP.** Comparison of rates of degradation of FtsZ, FtsZ(L378A), FtsZ(F377A), FtsZ(A376V) and FtsZ(P375G) in the presence and absence of GTP from in vitro degradation reactions containing 10 µM wild type or mutant fluorescent FtsZ and 1 µM ClpXP. Data from 3 replicates are presented as mean ± SEM.
(TIF)

Figure S2 **FtsZ mutant proteins with C-terminal mutations assemble into filaments.** FtsZ mutant proteins (5 µM) were incubated GTP, then visualized by negative staining and electron microscopy as described in *Experimental Procedures (SI)*. Arrows point to the appearance of filament pairs or bundles in micrographs showing FtsZ(352AAA), FtsZ(R379E) and FtsZ(352AAA, Δ_375-383_).
(TIF)

Figure S3 **MinC does not inhibit degradation of GFP-ssrA by ClpXP.** Degradation of GFP-ssrA (0.5 µM) by ClpXP (0.4 µM) was monitored as described in *Experimental Procedures* in the presence and absence of MinC (5 µM) by measuring the decrease of fluorescence over time.
(TIF)

Figure S4 **Mutations near the FtsZ C-terminal domain impair FtsZ function in vivo.** A. FtsZ mutant proteins were tested for function in vivo by monitoring high temperature growth of *ftsZ84* cells expressing FtsZ mutant proteins in a dilution spot plate assay under permissive (30 °C) and restrictive (42 °C) conditions. B. Z-ring localization of GFP-FtsZ mutant proteins was visualized by fluorescence microscopy in live cells (strain JC0390) undergoing division. Expression of GFP-FtsZ mutants proteins was induced by arabinose as described in *Experimental Procedures (SI)*. Images are representative of at least 3 data sets.
(TIF)

Figure S5 **Expression of GFP-tagged FtsZ mutant proteins causes Z-ring defects.** Z-ring localization of GFP-FtsZ wild type (WT) and mutant proteins was visualized by fluorescence microscopy (top panel) and DIC microscopy (bottom panel) in live cells (strain JC0390) undergoing division. Expression of GFP-FtsZ mutants proteins was induced by arabinose and cells were imaged as described in *Experimental Procedures (SI)*.
(TIF)

Text S1 **Experimental Procedures.**
(DOCX)

Table S1 ***E. coli*** **strains and plasmids used in functional assays in vivo.**
(PDF)
