# Supplementary figures and images for: Location of Dual Sites in E. coli FtsZ Important for Degradation by ClpXP; One at the C-Terminus and One in the Disordered Linker
Source: PLoS One. 2014 Apr 10;9(4):e94964. doi: 10.1371/journal.pone.0094964 (PMC3983244; doi:10.1371/journal.pone.0094964)

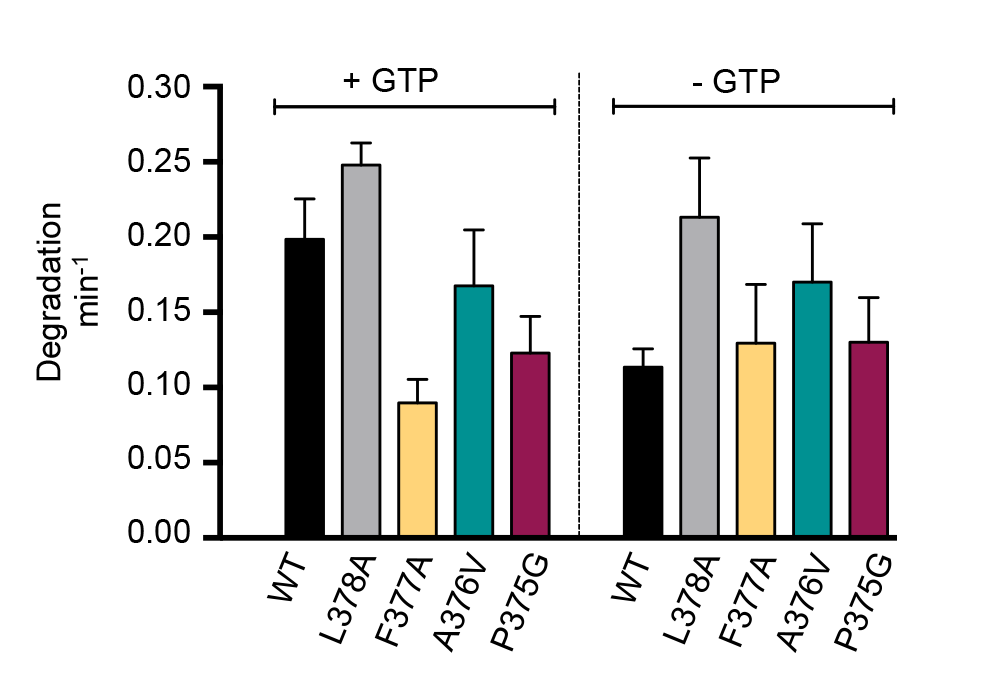

Supplement: Figure S1 — Substitution of residues near the FtsZ C-terminus modulates the rate of degradation by ClpXP. Comparison of rates of degradation of FtsZ, FtsZ(L378A), FtsZ(F377A), FtsZ(A376V) and FtsZ(P375G) in the presence and absence of GTP from in vitro degradation reactions containing 10 µM wild type or mutant fluorescent FtsZ and 1 µM ClpXP. Data from 3 replicates are presented as mean ± SEM. (TIF) [file pone.0094964.s001.tif]

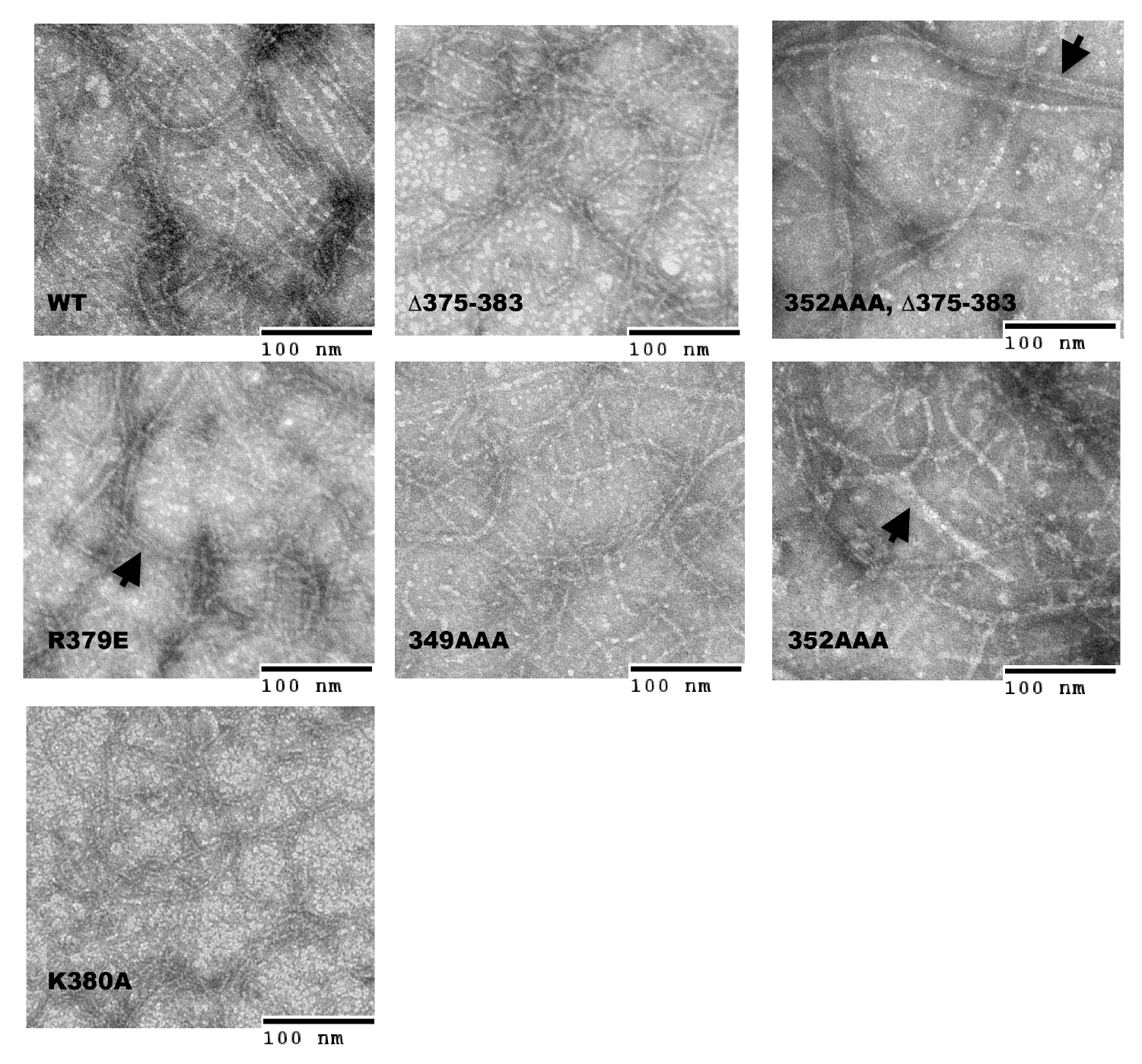

Supplement: Figure S2 — FtsZ mutant proteins with C-terminal mutations assemble into filaments. FtsZ mutant proteins (5 µM) were incubated GTP, then visualized by negative staining and electron microscopy as described in Experimental Procedures (SI). Arrows point to the appearance of filament pairs or bundles in micrographs showing FtsZ(352AAA), FtsZ(R379E) and FtsZ(352AAA, Δ375-383). (TIF) [file pone.0094964.s002.tif]

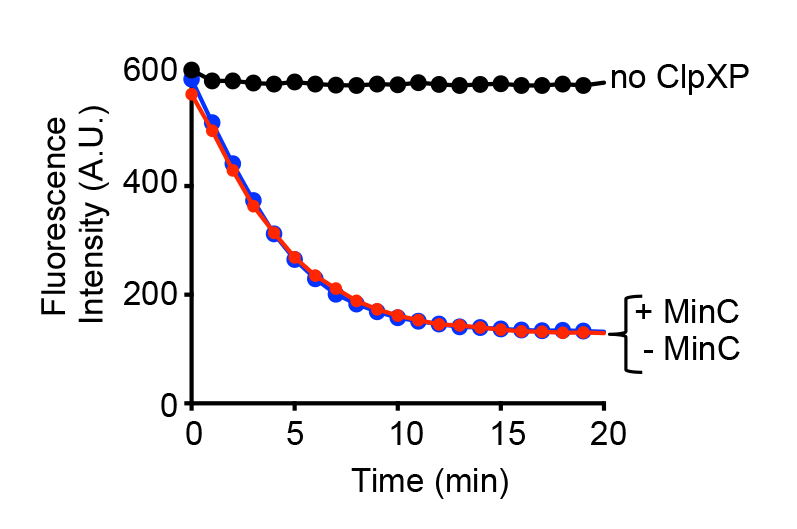

Supplement: Figure S3 — MinC does not inhibit degradation of GFP-ssrA by ClpXP. Degradation of GFP-ssrA (0.5 µM) by ClpXP (0.4 µM) was monitored as described in Experimental Procedures in the presence and absence of MinC (5 µM) by measuring the decrease of fluorescence over time. (TIF) [file pone.0094964.s003.tif]

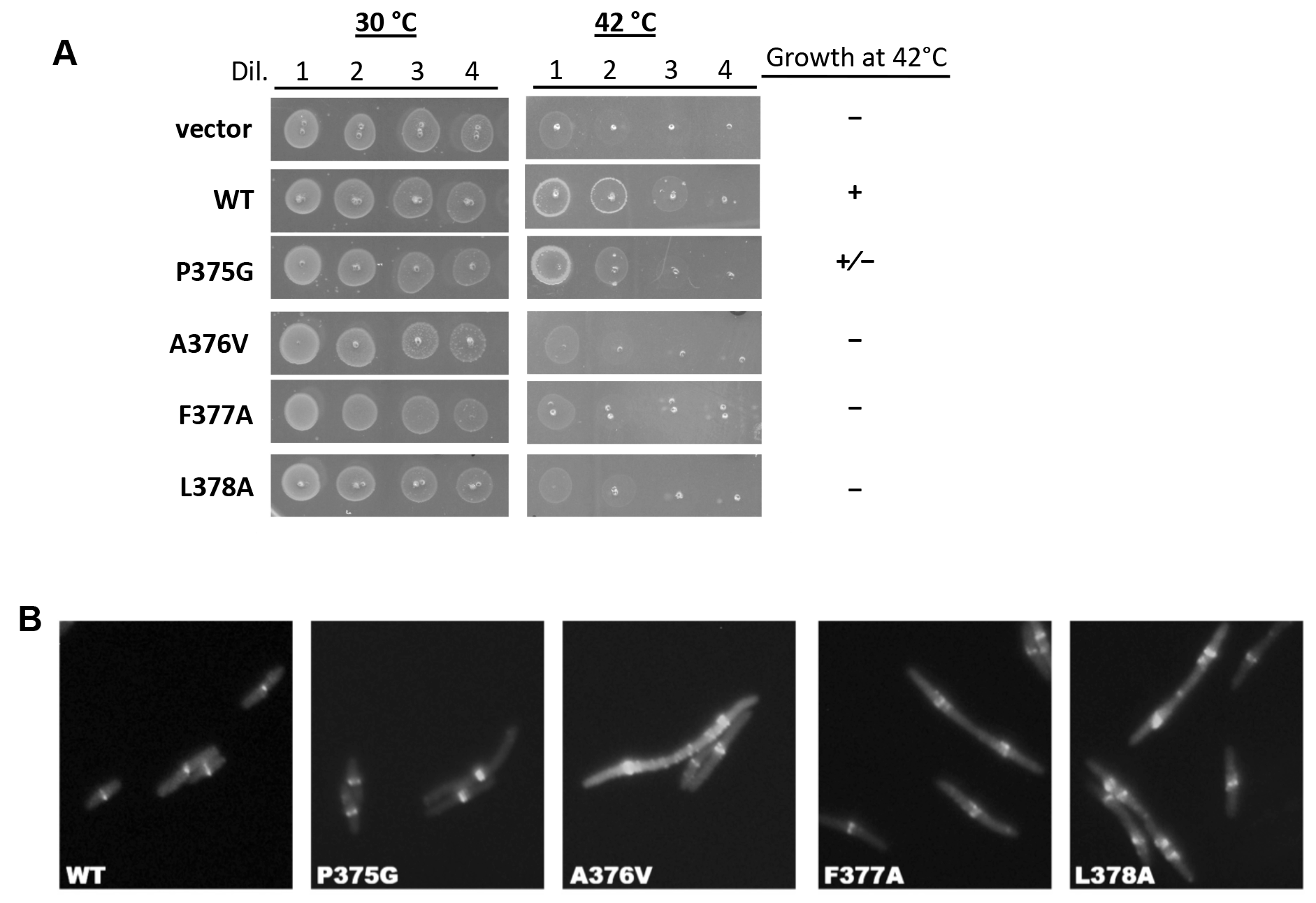

Supplement: Figure S4 — Mutations near the FtsZ C-terminal domain impair FtsZ function in vivo. A. FtsZ mutant proteins were tested for function in vivo by monitoring high temperature growth of ftsZ84 cells expressing FtsZ mutant proteins in a dilution spot plate assay under permissive (30 °C) and restrictive (42 °C) conditions. B. Z-ring localization of GFP-FtsZ mutant proteins was visualized by fluorescence microscopy in live cells (strain JC0390) undergoing division. Expression of GFP-FtsZ mutants proteins was induced by arabinose as described in Experimental Procedures (SI). Images are representative of at least 3 data sets. (TIF) [file pone.0094964.s004.tif]

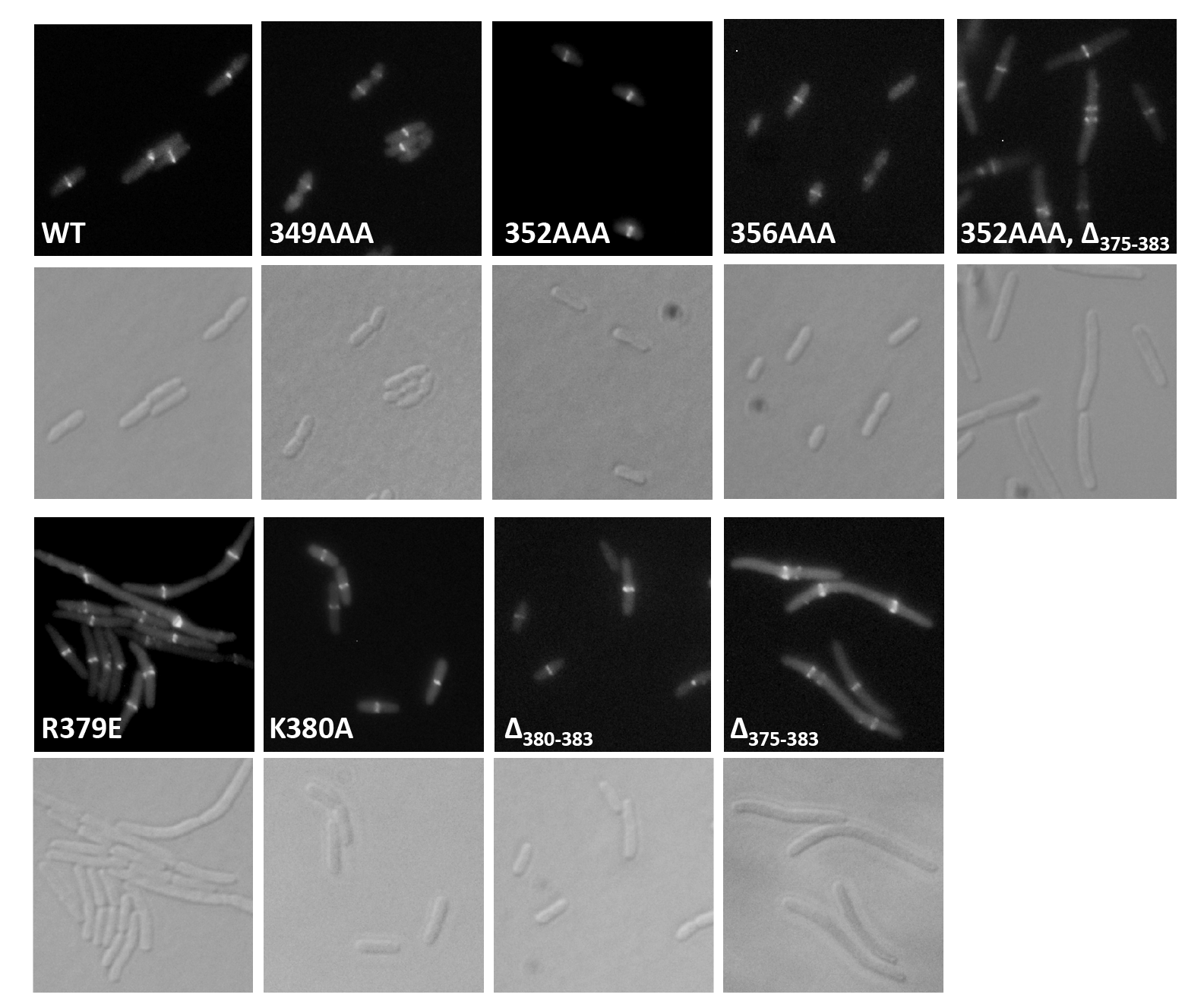

Supplement: Figure S5 — Expression of GFP-tagged FtsZ mutant proteins causes Z-ring defects. Z-ring localization of GFP-FtsZ wild type (WT) and mutant proteins was visualized by fluorescence microscopy (top panel) and DIC microscopy (bottom panel) in live cells (strain JC0390) undergoing division. Expression of GFP-FtsZ mutants proteins was induced by arabinose and cells were imaged as described in Experimental Procedures (SI). (TIF) [file pone.0094964.s005.tif]
