# Supplementary material for: Location of Dual Sites in E. coli FtsZ Important for Degradation by ClpXP; One at the C-Terminus and One in the Disordered Linker
Source: PLoS One. 2014 Apr 10;9(4):e94964. doi: 10.1371/journal.pone.0094964 (PMC3983244; doi:10.1371/journal.pone.0094964)
Supplement: Text S1 — Experimental Procedures. (DOCX) [file pone.0094964.s006.docx]

**Camberg, et al., “Location of dual sites in *E. coli* FtsZ important for degradation by ClpXP; one at the C-terminus and one in the disordered linker”**

**Supporting Information:**

**Experimental Procedures (SI)**

**Functional assays of FtsZ mutant proteins in vivo**

To observe the location of FtsZ rings in live cells, FtsZ wild type and mutant proteins were expressed as fusions to green fluorescent protein (GFP) in *E. coli* strain JC0390 (Table S1). Overnight cultures were diluted into fresh media containing arabinose (0.004%) and ampicillin (100 μg ml^−1^), then grown for two hours at 30°C. Cells were collected by centrifugation at 500 × *g* for 5 min, washed in phosphate-buffered saline (PBS) and pipetted onto thin 4% agarose pads containing MOPS medium supplemented with 0.5% glycerol. Cells were visualized with a Zeiss LSM 700 fluorescence microscope using the FITC filter. Images were processed using Adobe Photoshop CS6.

**Electron microscopy of FtsZ wild type and mutant polymers**

FtsZ wild type and mutant polymers were visualized by negative staining with uranyl acetate and electron microscopy as described [48]. Polymers were generated by addition of GTP (2 mM) to reactions containing 5 μM wild type or mutant FtsZ, in assembly buffer supplemented with 25 μg/ml acetate kinase and 15 mM acetyl phosphate.
