# Supplementary material for: Location of Dual Sites in E. coli FtsZ Important for Degradation by ClpXP; One at the C-Terminus and One in the Disordered Linker
Source: PLoS One. 2014 Apr 10;9(4):e94964. doi: 10.1371/journal.pone.0094964 (PMC3983244; doi:10.1371/journal.pone.0094964)
Supplement: Table S1 — E. coli strains and plasmids used in functional assays in vivo. (PDF) [file pone.0094964.s007.pdf]

TABLE S1. *E. coli* strains and plasmids used in functional assays in vivo

Strain or Plasmid	Genotype	Source, reference or Construction[a]
Strains		
BL21 (λDE3)	*F– ompT gal dcm lon hsdSB(rB- mB-) λ(DE3 [lacI lacUV5-T7 gene 1 ind1 sam7 nin5])*	EMD Millipore, USA
MG1655	*LAM- rph-1*	[23]
BW27750	*BW25113 DE(araFGH) (ΔaraEp kan PCP18-araE)*	[22]
JC0390	MG1655 *(ΔaraEp kan PCP18-araE)*	P1(BW27750) x MG1655
MCZ84	*ftsZ84(ts), leu-260::Tn10, [araD139], Δ(argF-lac)169, LAM-, e14-, flhD5301, ΔfruK-yeiR)725(fruA), relA1, rpsL150(strR), rbsR22, Δ(fimB-fimE)632(::IS1), deoC1*	[41]
Plasmids		
pBAD24	*amp* (expression vector)	[24]
pBAD-FtsZ	*amp* P_{ara}::*ftsZ*	[25]
pBAD-FtsZ(P375G)	*amp* P_{ara}::*ftsZ(P375G)*	This study
pBAD-FtsZ(A376V)	*amp* P_{ara}::*ftsZ(A376V)*	This study
pBAD-FtsZ(F377A)	*amp* P_{ara}::*ftsZ(F377A)*	This study
pBAD-FtsZ(L378A)	*amp* P_{ara}::*ftsZ(L378A)*	This study
pBAD-FtsZ(R379E)	*amp* P_{ara}::*ftsZ(R379E)*	This study
pBAD-FtsZ(K380A)	*amp* P_{ara}::*ftsZ(K380A)*	This study
pBAD-FtsZ($\Delta_{380\text{-}383}$)	*amp* P_{ara}::*ftsZ*($\Delta_{380\text{-}383}$)	This study
pBAD-FtsZ($\Delta_{375\text{-}383}$)	*amp* P_{ara}::*ftsZ*($\Delta_{375\text{-}383}$)	This study
pBAD-FtsZ(349AAA)	*amp* P_{ara}::*ftsZ(349AAA)*	This study
pBAD-FtsZ(352AAA)	*amp* P_{ara}::*ftsZ(352AAA)*	This study
pBAD-FtsZ(356AAA)	*amp* P_{ara}::*ftsZ(356AAA)*	This study
pBAD-FtsZ(352AAA, $\Delta_{375\text{-}383}$)	*amp* P_{ara}::*ftsZ(352AAA,* $\Delta_{375\text{-}383}$)	This study
pGfp-FtsZ	*amp* P_{ara}::*gfp-ftsZ*	[25]
pGfp-FtsZ(P375G)	*amp* P_{ara}::*gfp-ftsZ(P375G)*	This study
pGfp-FtsZ(A376V)	*amp* P_{ara}::*gfp-ftsZ(A376V)*	This study
pGfp-FtsZ(F377A)	*amp* P_{ara}::*gfp-ftsZ(F377A)*	This study
pGfp-FtsZ(L378A)	*amp* P_{ara}::*gfp-ftsZ(L378A)*	This study
pGfp-FtsZ(R379E)	*amp* P_{ara}::*gfp-ftsZ(R379E)*	This study
pGfp-FtsZ(K380A)	*amp* P_{ara}::*gfp-ftsZ(K380A)*	This study
pGfp-FtsZ($\Delta_{380\text{-}383}$)	*amp* P_{ara}::*gfp-ftsZ*($\Delta_{380\text{-}383}$)	This study
pGfp-FtsZ($\Delta_{375\text{-}383}$)	*amp* P_{ara}::*gfp-ftsZ*($\Delta_{375\text{-}383}$)	This study
pGfp-FtsZ(349AAA)	*amp* P_{ara}::*gfp-ftsZ(349AAA)*	This study
pGfp-FtsZ(352AAA)	*amp* P_{ara}::*gfp-ftsZ(352AAA)*	This study
pGfp-FtsZ(356AAA)	*amp* P_{ara}::*gfp-ftsZ(356AAA)*	This study
pGfp-FtsZ(352AAA, $\Delta_{375\text{-}383}$)	*amp* P_{ara}::*gfp-ftsZ(352AAA,* $\Delta_{375\text{-}383}$)	This study

[a] Strain constructions by P1 transduction are described as the following: P1(donor) x recipient.
